# Nearly massless Dirac fermions hosted by Sb square net in BaMnSb_2_

**DOI:** 10.1038/srep30525

**Published:** 2016-07-28

**Authors:** Jinyu Liu, Jin Hu, Huibo Cao, Yanglin Zhu, Alyssa Chuang, D. Graf, D. J. Adams, S. M. A. Radmanesh, L. Spinu, I. Chiorescu, Zhiqiang Mao

**Affiliations:** 1 Department of Physics and Engineering Physics, Tulane University, New Orleans, LA 70018, USA; 2 Quantum Condensed Matter Division, Oak Ridge National Laboratory, TN 37830, USA; 3 National High Magnetic Field Lab, Tallahassee, FL 32310, USA; 4 Department of Physics and Advanced Materials Research Institute, University of New Orleans, New Orleans, LA 70148, USA; 5 Department of Physics, Florida State University, Tallahassee, FL 32306, USA.

## Abstract

Layered compounds AMnBi_2_ (A = Ca, Sr, Ba, or rare earth element) have been established as Dirac materials. Dirac electrons generated by the two-dimensional (2D) Bi square net in these materials are normally massive due to the presence of a spin-orbital coupling (SOC) induced gap at Dirac nodes. Here we report that the Sb square net in an isostructural compound BaMnSb_2_ can host nearly massless Dirac fermions. We observed strong Shubnikov-de Haas (SdH) oscillations in this material. From the analyses of the SdH oscillations, we find key signatures of Dirac fermions, including light effective mass (~0.052*m*_0_; *m*_0_, mass of free electron), high quantum mobility (1280 cm^2^V^−1^S^−1^) and a π Berry phase accumulated along cyclotron orbit. Compared with AMnBi_2_, BaMnSb_2_ also exhibits much more significant quasi two-dimensional (2D) electronic structure, with the out-of-plane transport showing nonmetallic conduction below 120 K and the ratio of the out-of-plane and in-plane resistivity reaching ~670. Additionally, BaMnSb_2_ also exhibits a G-type antiferromagnetic order below 283 K. The combination of nearly massless Dirac fermions on quasi-2D planes with a magnetic order makes BaMnSb_2_ an intriguing platform for seeking novel exotic phenomena of massless Dirac electrons.

Three-dimensional topological semimetals, including Dirac semimetals (DSMs)^1^,^2^,^3^,^4^,^5^,^6^, Weyl semimetals (WSMs)^7^,^8^,^9^,^10^,^11^,^12^,^13^,^14^,^15^ and Dirac nodal-line semimetals^16^,^17^,^18^,^19^,^20^,^21^ represent new quantum states of matter and have stimulated intensive studies. These materials possess bulk relativistic quasiparticles with linear energy-momentum dispersion. DSMs feature linear band crossings at discrete Dirac nodes. In WSMs, the Weyl nodes with opposite chirality appear in pairs, and each pair of Weyl nodes can be viewed as evolving from the splitting of Dirac node due to the lifted spin degeneracy arising from either broken spatial inversion symmetry or broken time reversal symmetry (TRS). The linear band dispersions in these materials are topologically protected by crystal symmetry and lead to many distinct physical properties such as large linear magnetoresistance and high bulk carrier mobility^22^. WSMs also show exotic surface “Fermi arc” connecting a pair of Weyl nodes of the opposite chirality^7^,^8^. These exotic properties of topological semimetals have potential applications in technology.

AMnBi_2_ (A = alkali earth/rare earth metal) is one of the established Dirac semimetals^23^,^24^,^25^,^26^,^27^,^28^,^29^,^30^,^31^,^32^,^33^. These materials share common structure characteristics, consisting of alternately stacked MnBi4 tetrahedral layers and A-Bi-A sandwich layers^23^,^26^,^27^,^29^,^31^,^33^. In an A-Bi-A sandwich layer, Bi atoms form a square net and harbor Dirac fermions, with coincident (e.g. SrMnBi_2_^34^) or staggered (e.g. CaMnBi_2_^35^) stacking of A atoms above and below the Bi plane. In the Mn centered edge sharing MnBi4 tetrahedral layers, antiferromagnetic (AFM) order usually develops near room temperature^36^,^37^ and such layers are expected to be less conducting^23^,^27^. Dirac fermions in AMnBi_2_ have been found to interplay with magnetism, leading to novel exotic properties. This has been demonstrated in YbMnBi_2_^15^ and EuMnBi_2_^31^. Evidence for Weyl state has been observed in YbMnBi_2_, which has been proposed to be caused by the TRS breaking due to the ferromagnetic (FM) component of a canted AFM state^15^. In EuMnBi_2_, half-integer bulk quantum Hall Effect (QHE) occurs due to the magnetic order induced two-dimensional (2D) confinement of Dirac fermions^31^.

One disadvantage of AMnBi_2_ as Dirac semimetals is that the strong spin orbit coupling (SOC) due to heavy Bi atoms opens gap at Dirac nodes^23^,^38^, leading to massive Dirac electrons. For instance, in SrMnBi_2_, the SOC-induced gap at the Dirac node is about 40 meV^23^ and the effective mass of Dirac fermions estimated from the analyses of Shubnikov-de Haas (SdH) oscillations is ~0.29*m*_0_ (*m*_0_, the mass of free electron)^23^, much heavier than the Dirac fermions in the 3D gapless DSM Cd_3_As_2_ where m^*^ ~0.02–0.05*m*_0_^39^,^40^,^41^,^42^. Therefore, one possible route to realize massless Dirac fermions in AMnBi_2_-type material is to replace Bi with other lighter main group elements such as Sb and Sn, whose SOC effect is much weaker. Under this motivation, we previously studied SrMnSb_2_^43^ and found the 2D Sb layer can indeed harbor much lighter relativistic fermions with m* ~0.14*m*_0_. Moreover, this material shows FM properties: the Mn sublattice develops a FM order for 304 K < T < 565 K, but a canted AFM state with a FM component for T < 304 K. Coupling between ferromagnetism and quantum transport properties of relativistic fermions has also been observed^43^. These interesting results further motivated us to investigate the isostructural compound BaMnSb_2_, the material studied in this work.

BaMnSb_2_ crystalizes in a tetragonal structure with the space group of *I4/mmm*^34^, similar to the structure of SrMnBi_2_ but different from the orthorhombic structure of SrMnSb_2_^43^. The orthorhombic distortion in SrMnSb_2_ leads Sb atoms in the Sr-Sb-Sr sandwich layer to form zig-zag chains. However, in BaMnSb_2_, as Ba has larger ionic radius than Sr, the stronger interaction between Ba atoms and Sb atoms suppresses orthorhombic distortion^38^ and lead to a Sb square net lattice (Fig. 1a), which is an analogue of the Bi square net in SrMnBi_2_. In addition, the Ba layers are coincidently stacked along the Sb layer in BaMnSb_2_, which is also distinct from the staggeredly arranged Sr atoms in SrMnSb_2_^38^. First principle calculations have predicted that BaMnSb_2_ exhibits Dirac fermion behavior and its SOC induced gap near the Dirac node is as small as ~20 meV, about half of the gap in SrMnBi_2_^38^.

In this paper, we will show BaMnSb_2_ indeed is a Dirac material with the Dirac node being very close to the Fermi level. Through the analyses of SdH oscillations, we find this material hosts nearly massless Dirac fermions (m^*^ ~0.052*m*_0_). As compared with AMnBi_2_ and SrMnSb_2_, BaMnSb_2_ possesses the smallest Fermi surface (FS) with the most significant 2D character. Additionally, BaMnSb_2_ also exhibits a G-type AFM order below 283 K. These findings suggest that BaMnSb_2_ is a promising candidate for seeking novel exotic phenomena of massless Dirac fermions.

## Results and Discussions

The BaMnSb_2_ single crystals used in this study were synthesized using self-flux method (see Method). The composition measured by an energy dispersive X-ray spectrometer (EDS) can be expressed as Ba_1-y_Mn_1-z_Sb_2_, with *y* < 0.05 and 0.05 < *z* < 0.12. The nonstoichiometry of Sr and Mn was also found in SrMnSb_2_ where the actual composition measured by EDS can be described by Sr_1-y_Mn_1-z_Sb_2_ (*y*, *z* < 0.1)^43^. The neutron diffraction experiment on a piece of single crystal with the measured composition of Ba_0.96_Mn_0.94_Sb_2_ confirms the tetragonal structure with the space group *I4/mmm* reported by Cordier & Schafer^34^. The lattice parameters and atomic positions extracted from the structural confinement are summarized in Table 1. We note that the lattice constant *c* = 23.85 Å obtained from our structural refinement is smaller than the previously-reported value of 24.34 Å^34^, which may be attributed to the deficiencies of Ba and Mn in our sample.

**Figure 1:**
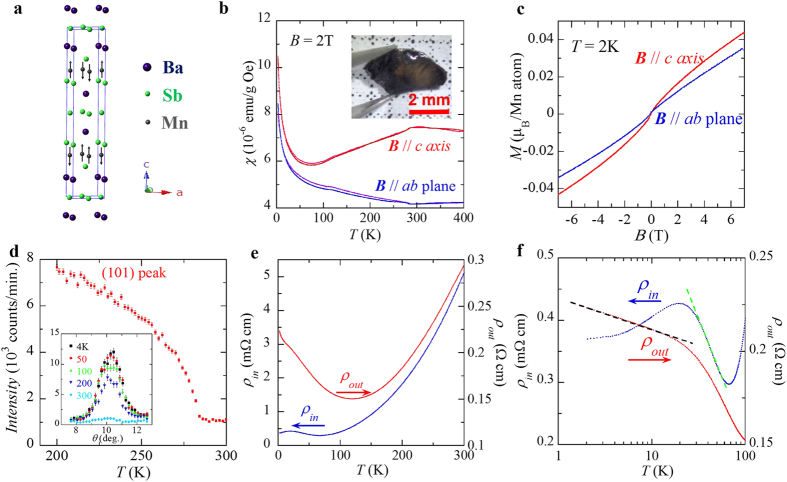
Crystal strucutre, magnetic and transport properties of BaMnSb_2_. (**a**) Crystal and magnetic structure of BaMnSb_2_. (**b)** Susceptibility as a function of temperature measured with a 2T magnetic field applied along the *c* axis (χ_c_) and along the *ab* plane (χ_ab_) under zero field cooling (ZFC) and field cooling (FC) histories. Red: χ_c_ of FC; dark red: χ_c_ of ZFC; purple: χ_ab_ of FC; blue: χ_ab_ of ZFC. Inset in (**b**), an optical image of a typical BaMnSb_2_ single crystal. (**c)** Isothermal magnetization along the *c* axis (red) and along the *ab* plane (blue). (**d)** Temperature dependence of the Bragg peak intensity at (101) indicates the magnetic order below 283 K. Inset in (**d**), the Bragg peak (101) scanned at the selected temperatures. (**e)** In-plane resistivity (ρ_*in*_) and out-of-plane resistivity (ρ_*out*_) as a function of temperature under zero magnetic field. (**f**) ρ_*in*_ and ρ_*out*_ plotted on logarithmic scale.

From magnetic susceptibility measurements on BaMnSb_2_ single crystals, we have observed signatures of antiferromagnetism. As shown in Fig. 1b, at temperatures below 285 K, the susceptibility *χ* exhibits a striking decrease for the magnetic field applied along the c-axis (B//c), but a clear upturn for in-plane field (B//ab). As the temperature is lowered below 50 K, *χ* displays sharp upturns for both field orientations. Similar features have been observed in AMnBi_2_ (A = Ca, Sr, and Ba)^23^,^25^,^26^,^32^,^33^,^36^. Neutron scattering studies on CaMnBi_2_ and SrMnBi_2_ have demonstrated that those magnetic anomalies probed in susceptibility originate from an AFM order formed on the Mn-sublattice^36^. Given the similar behavior in susceptibility between BaMnSb_2_ and AMnBi_2_, we can reasonably attribute the magnetic transition at 285 K seen in BaMnSb_2_ to an AFM transition. We note BaMnSb_2_ exhibits distinct magnetic anisotropy from AMnBi_2_. As seen in Fig. 1b, *χ* of B//c (*χ*_*c*_) is much larger than *χ* of B//ab (*χ*_*ab*_) at temperatures both above and below T_N_. However, in AMnBi_2_, *χ*_*ab*_ is almost equal to *χ*_*c*_ for *T* ≥ *T*_*N*_, but > *χ*_*c*_ for *T* < *T*_*N*_^23^,^26^,^32^. Additionally, the isothermal magnetization *M*(*H*) of BaMnSb_2_ (Fig. 1c) also looks different from that of AMnBi_2_. We have observed weak FM polarization behavior (Fig. 1c), in contrast with the nearly linear field dependence of *M* for BaMnBi_2_^32^. These discrepancies imply that BaMnSb_2_ and AMnBi_2_ may not share an identical magnetic structure.

Magnetic structures of CaMnBi_2_, SrMnBi_2_ and YbMnBi_2_ have been determined from neutron scattering experiment^36^,^37^. Although these materials show similar Néel-type AFM coupling within the plane and the moments are oriented along the c-axis, their interlayer coupling is different. CaMnBi_2_ (space group *P4/nmm*) and YbMnBi_2_ (*P4/nmm*) features FM interlayer coupling, whereas SrMnBi_2_ (*I4/mmm*) is characterized by interlayer AFM coupling^36^,^37^. The magnetic structure of BaMnSb_2_ determined from our neutron scattering experiments is similar to that of SrMnBi_2_, *i.e.* both the interlayer and intralayer couplings between two nearest moments are AFM, which is also called the nearest neighbor G-type AFM order. The Néel temperature probed in the neutron scattering experiment is ~283 K, as shown in Fig. 1d which shows the Bragg peak at (101) (dominated by the magnetic scattering, see the inset) as well as the temperature dependence of the (101) Bragg peak intensity. The ordered moment of Mn at 4 K estimated from the magnetic structure refinement at 4 K is 3.950(85) μ_*B*_/Mn, comparable to the ordered moments probed in CaMnBi_2_, SrMnBi_2_, YbMnBi_2_ and Sr_1-y_Mn_1-z_Sb_2_^36^,^37^,^43^. As indicated above, the isothermal magnetization of BaMnSb_2_ exhibits weak FM polarization (Fig. 1c). This feature, together with the upturn of *χ*_*ab*_ below *T*_*N*_, sharp upturns of *χ*_*ab*_ and *χ*_*c*_ below 50 K, and the small irreversibility of *χ*_*ab*_ between field cooling (FC) and zero-field-cooling (ZFC) measurements, is reminiscent of a canted AFM state with a FM component. However, moment canting is generally not expected for a tetragonal structure for symmetry considerations. If the weak ferromagnetism turns out to be intrinsic for BaMnSb_2_, the possible origin may be associated with its actual nonstoichiometric composition Ba_1-y_Mn_1-z_Sb_2_ as mentioned above. In our previous studies on orthorhombic SrMnSb_2_^43^, we have demonstrated a FM component arising from a canted AFM state; the saturated FM moment sensitively depends on Sr and Mn deficiencies, ranging from 0.6 μ_B_/Mn to 0.005μ_B_/Mn. With this in mind, we can speculate that Ba and Mn deficiencies possibly lead to local orthorhombic distortion, thus resulting in local canted AFM states. However, we have to point out that small FM components cannot be resolved in neutron scattering experiments. Hence it is not surprising to see the absence of FM response in our neutron scattering experiment.

We have also characterized the electronic transport properties of BaMnSb_2_ single crystals. In Fig. 1e we present both in-plane (*ρ*_*in*_) and out-of-plane (*ρ*_*out*_) resistivity as a function of temperature, from which we found several signatures distinct from that of (Ca/Sr/Ba)MnBi_2_^26^,^30^,^32^,^33^. First, BaMnSb_2_ shows much stronger electronic anisotropy than (Ca/Sr/Ba)MnBi_2_, which is manifested in its larger *ρ*_*out*_/*ρ*_*in*_ ratio. The *ρ*_*out*_/*ρ*_*in*_ ratio at 2 K ranges from 15 to 100 for (Ca/Sr/Ba)MnBi_2_^26^,^30^,^32^,^33^, but rises to 670 for BaMnSb_2_, which is comparable to the value of *ρ*_out_/*ρ*_in_ (~609) seen in SrMnSb_2_^43^. Such a large electronic anisotropy of BaMnSb_2_ suggests its electronic structure is quasi-2D like, which is further confirmed in our measurements of angular dependence of SdH oscillation frequency as shown below. Second, unlike (Ca/Sr/Ba)MnBi_2_ whose *ρ*_*out*_(*T*) always exhibits a hump due to a crossover from high-temperature incoherent to low-temperature coherent conduction^26^,^30^,^32^,^33^, BaMnSb_2_ displays an opposite behavior in *ρ*_*out*_(*T*) (Fig. 1e); a crossover from high-temperature metallic conduction to low-temperature localization is observed, which leads to a broad minimum in *ρ*_*out*_(*T*) around 120 K. The temperature dependence of in-plane resistivity *ρ*_*in*_(*T*) of BaMnSb_2_ also differs from that of (Ca/Sr/Ba)MnBi_2_. (Ca/Sr/Ba)MnBi_2_ features a quadratic temperature dependence for *ρ*_*in*_ in low temperature range^23^,^26^,^32^,^33^, while *ρ*_*in*_ of BaMnSb_2_ exhibits localization behavior below 80 K but crossovers to metallic behavior below 11 K. These differences imply the transport mechanism in BaMnSb_2_ is somewhat different from that in (Ca/Sr/Ba)MnBi_2_. We note that in the temperature region where the localization behavior occurs, both *ρ*_*out*_(*T*) and *ρ*_*in*_(*T*) follow a logarithmic temperature dependence, as denoted by the dashed lines in Fig. 1f which presents *ρ*_*out*_(*T*) and *ρ*_*in*_(*T*) on the log*T* scale. This observation is reminiscent of Kondo effect. Given that we have an AFM lattice formed from local moments of Mn ions, the presence of Kondo effect is possible in principle. But, this naturally leads to a question why such an effect occurs only to BaMnSb_2_, but not to (Ca/Sr/Ba)MnBi_2_ and SrMnSb_2_ with similar AFM lattices. Clear understanding of this issue requires further studies, but one possible interpretation is that the Kondo effect depends on the dimensionality of electronic structure, and may be enhanced in BaMnSb_2_ due to its highly 2D electronic structure. Moreover, we would like to point out the localization behavior seen in BaMnSb_2_ cannot be attributed to disorder induced localization since in Sr_1-y_Mn_1-z_Sb_2_ with the level of disorders being comparable or higher than that of BaMnSb_2_, no localization behavior is observed^43^.

**Figure 2:**
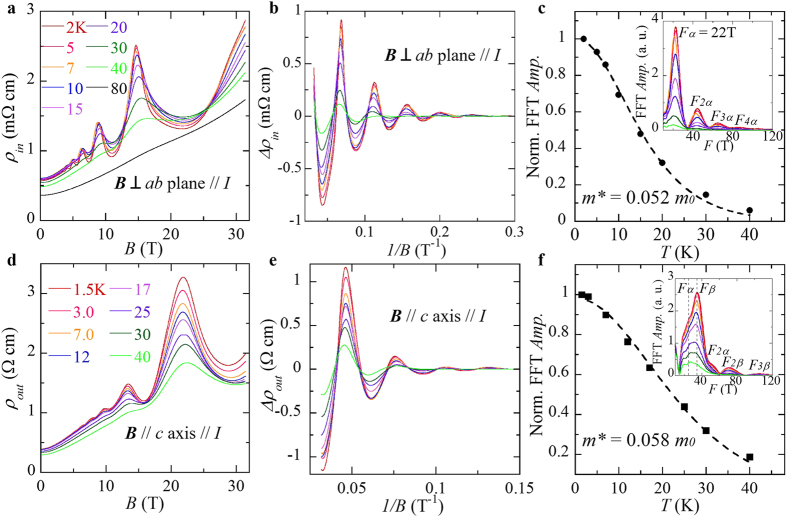
Quantum transport properties of BaMnSb_2_. (**a)** The in-plane resistivity, ρ_*in*_, as a function of field up to 31T at different temperatures. (**b)** The oscillatory component of ρ_*in*_vs. 1/B at different temperatures. (**c**) The temperature dependence of the normalized FFT amplitude. The dashed line curve is the fit to the Lifshitz-Kosevich (LK) formula from 2 to 40 K. Inset: FFT spectra of Δ*ρ*_*in*_(*B*) at different temperatures (the FFT was done in the field range of 3T–31T). (**d)** The out-of-plane resistivity, ρ_*out*_. as a function of field at different temperatures. (**e)** The oscillatory component of ρ_*out*_ vs. 1/B. (**f)** The temperature dependence of the normalized FFT amplitude. The dashed line is the fit to the LK formula. Inset: FFT spectra of Δ*ρ*_*out*_(*B*) at different temperatures (the FFT was done in the field range of 5T–31T).

Like (Ca/Sr/Ba)MnBi_2_ and SrMnSb_2_, BaMnSb_2_ also exhibits quantum transport properties as revealed by our magnetotransport measurements, In Fig. 2a,d, we present the field dependences of in-plane (*ρ*_*in*_) and out-of-plane (*ρ*_*out*_) resistivity measured at various temperatures for BaMnSb_2_, respectively. Strong SdH oscillations, which sustain up to above 40 K, are observed in both *ρ*_*in*_(*B*) and *ρ*_*out*_(*B*). In Fig. 2b,e we present the oscillatory components of *ρ*_*in*_ and *ρ*_*out*_, respectively. From Fast Fourier Transformation (FFT) analyses of Δ*ρ*_*in*_ and Δ*ρ*_*out*_ (see the insets to Fig. 2c,f), we find that the SdH oscillations of ρ_*in*_ consists of a single frequency (~22T), whereas the oscillations of ρ_*out*_ include two frequencies (*i.e. F*_α_~25T and *F*_β_ ~35T). Such a difference is likely caused by the non-stoichiometric composition. As mentioned above, the actual composition of our BaMnSb_2_ crystals involves Ba and Mn non-stoichiometry, which could lead to slight modification for electronic structure in different samples. To verify this speculation, we have measured many samples and find that their oscillation frequencies indeed show variation, ranging from 20T to 35T. Given that the quantum oscillation frequency is directly linked to the extremal Fermi surface cross-section area *A*_*F*_ by the Onsager relation *F* = (*Φ*_0_/2π^2^)*A*_*F*_, a small oscillation frequency is generally expected for topological semimetals with the Dirac node being near the Fermi level. We note that the quantum oscillation frequency of 22T probed in our BaMnSb_2_ crystals is the smallest as compared with AMnBi_2_ and SrMnSb_2_, implying that if BaMnSb_2_ turns out to be a Dirac material, its Dirac band crossing points must be very close to the Fermi level.

Evidence for Dirac fermions in BaMnSb_2_ has been obtained from the further analyses of the SdH oscillations. As shown in Fig. 2c,f, the effective cyclotron mass m^*^ can be extracted from the fit of the temperature dependence of the normalized FFT peak amplitude to the thermal damping factor of Lifshitz-Kosevich (LK) equation^44^, *i.e*. 
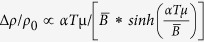
, where *ρ*_*0*_ is the zero field resistivity, and *α* = (2π^2^*k*_B_*m*_0_)/(*ħe*). μ is the ratio of effective mass of cyclotron motion to the free electron mass. 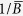 is the average inverse field for FFT analysis. We did the FFT within the field 3T–31T range for ρ_*in*_ and the 5T–31T range for ρ_*out*_, with 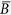 being 5.47T and 8.61T respectively. As seen in Fig. 2c,f, the best fits yield m* = 0.052*m*_0_ and 0.058*m*_0_, respectively, for the SdH oscillations of ρ_*in*_ and ρ_*out*_. For *ρ*_*out*_, the fit was performed for the component with the oscillation frequency of 35T, whose FFT peak can be clearly resolved. The effective mass of m* = 0.052*m*_0_ and 0.058*m*_0_ seen in BaMnSb_2_ is much smaller than that of other known AMnBi_2_^23^,^26^,^32^,^37^ and SrMnSb_2_^43^, but comparable to that of gapless Dirac semimetal Cd_3_As_2_^39^,^40^,^41^,^42^. Detailed comparisons of m* as well as other parameters derived from SdH oscillations are shown in Table 2.

**Figure 3:**
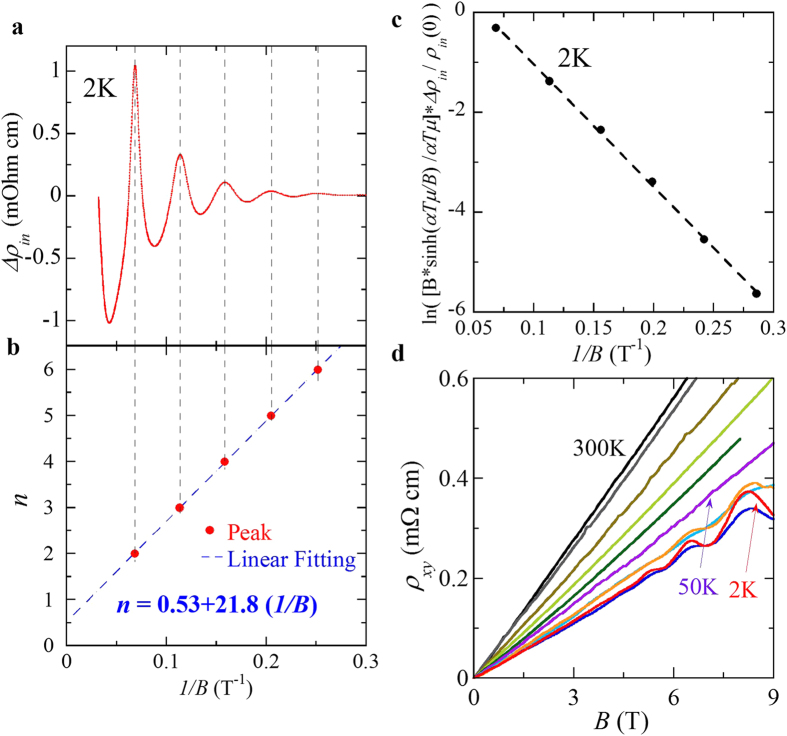
Berry phase, quantum mobility and Hall resistivity of BaMnSb_2_. (**a)** The oscillatory component of in-plane resistivity, ρ_*in*_, vs. 1/B at 2 K. As the longitudinal resistivity (ρ_*in*_ = ρ_*xx*_) is much larger than transverse resistivity (ρ_xy_), integer Landau level (LL) indices are assigned to the maximum of resistivity (see text). (**b)** LL fan diagram. The blue dashed line represents the linear fit. **c,** Dingle plot for the in-plane quantum oscillations Δρ_*in*_ at 2 K. (**d)** Hall resistivitivity as a function of field at various temperatures (*T* = 2, 5, 10, 20, 50, 100, 150, 200, 250 and 300 K).

To further verify if the nearly massless electrons in BaMnSb_2_ is of topological nature of Dirac fermions, we extracted the Berry phase accumulated along cyclotron orbit from the analyses of SdH oscillations. Berry phase should be zero for a non-relativistic system with parabolic band dispersion, while a finite value up to π is expected for Dirac fermions^45^,^46^. We present the Landau level (LL) fan diagram constructed from the SdH oscillations of *ρ*_*in*_ for BaMnSb_2_ in Fig. 3a,b, where integer LL indices are assigned to the maxima of *ρ*_*in*_. Our definition of LL index is based on the customary practice that integer LL indices are assigned to the minima of conductivity^46^,^47^. In-plane conductivity *σ*_*xx*_ can be converted from the longitudinal resistivity *ρ*_*xx*_ and the transverse (Hall) resistivity *ρ*_*xy*_ using *σ*_*xx*_ = *ρ*_*xx*_*/*(*ρ*_*xx*_^2^ + *ρ*_*xy*_^2^). Since our measured *ρ*_*xy*_ (Fig. 3d) is about 1/3–1/4 of *ρ*_*xx*_ (Fig. 2a) for *B* < 9 T, *σ*_*xx*_ ≈*1/ρ*_*xx*_, which justifies our definition of LL index. As seen in Fig. 3b, the intercept on the LL index axis obtained from the extrapolation of the linear fit in the fan diagram is 0.53, very close to the expected value of 0.5 for a 2D Dirac system with a π Berry phase. The oscillation frequency derived from the fit is 21.8T, nearly the same as the frequency obtained from the FFT analyses of the SdH oscillations of *ρ*_*in*_ (see the inset to Fig. 2c), suggesting that our linear fit in the fan diagram is reliable^46^. The Berry phase derived from the above fan diagram analyses clearly indicates that the nearly massless electrons probed in the SdH oscillations are Dirac fermions.

Dirac Fermions are usually characterized by high quantum mobility, as seen in Cd_3_As_2_^22^. This is also seen in BaMnSb_2_. The quantum mobility is directly related with the quantum relaxation time *τ*_*q*_ by *μ*_*q*_ = *eτ*_*q*_/m*. *τ*_*q*_ characterizes quantum life time, the time scale over which a quasiparticle stays in a certain eigenstate. *τ*_*q*_ can be found from the field damping of quantum oscillation amplitude, *i.e.*, 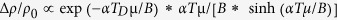. *T*_*D*_ is the Dingle temperature and is linked with *τ*_*q*_ by *T*_D_ = *ħ*/(2π*k*_B_*τ*_*q*_). With m^*^ being the known parameter, *τ*_*q*_ at *T* = 2 K can be extracted through the linear fit of ln ([B* sinh (*αTμ*/*B*)/*αTμ*]*Δ*ρ*/*ρ*_*0*_) against 1/B. As shown in Fig. 3c, we have obtained *τ*_*q*_ = 3.8 × 10^−14^ s, from which the quantum mobility *μ*_*q*_(=*eτ*_*q*_/m^*^) is estimated to be 1280 cm^2^V^−1^s^−1^, much higher than that of SrMnBi_2_ (250 cm^2^V^−1^s^−1^ 23) or SrMnSb_2_ (~570 cm^2^V^−1^s^−1^ 43) (see Table 2). In general, the transport mobility is one or two orders of magnitude higher than quantum mobility, since the transport mobility is sensitive only to large angle scattering of carriers, while the quantum mobility is sensitive to both small and large angle scatterings. However, this was not observed in BaMnSb_2_. Using the Hall coefficient *R*_H_ data extracted from Hall resistivity data shown in Fig. 3d, the transport mobility *μ*_tr_(=*R*_*H*_/*ρ*_*xx*_) at 1.8 K is estimated to be ~1300 cm^2^V^−1^S^−1^ for BaMnSb_2_, much less than that of SrMnSb_2_ (*μ*_tr_ ~12500 cm^2^V^−1^S^−1^) at low temperature^43^. The low *μ*_tr_ in BaMnSb_2_ may be associated with the transport localization behavior seen in *ρ*_out_ and *ρ*_in_.

**Figure 4:**
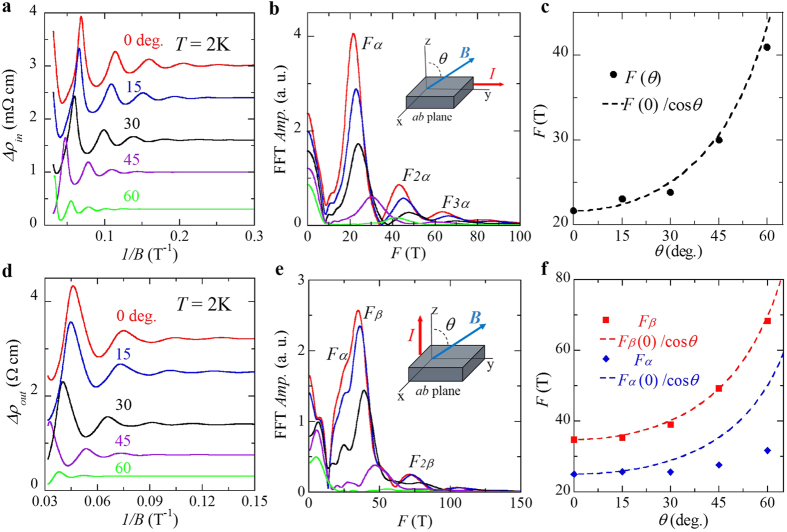
Angular dependences of SdH oscillations and the oscillation frequencies for BaMnSb_2_. (**a)** The oscillatory component of in-plane resistivity, Δρ_*in*_, vs. 1/B measured under different field orientations. The data has been shifted for clarity. (**b)** The FFT spectra of Δ*ρ*_*in*_(*B*) at different field orientations. Inset: the diagram of the measurement setup; θ is defined as the angle between the magnetic field and the out-of-plane direction. (**c**) The angle dependence of SdH oscillation frequency determined from the FFT of Δ*ρ*_*in*_(*B*). The dashed curve is the fit to *F*(θ) = *F*(0)/cosθ. (**d**) The oscillatory component of out-of-plane resistivity, Δρ_*out*_. vs. 1/B measured under different field orientations. The data has been shifted for clarity. (**e)** The FFT spectra of Δ*ρ*_*out*_(*B*) at different field orientations. Inset: the diagram of the measurement setup. (**f)** The angular dependence of SdH oscillation frequency determined from the FFT of Δ*ρ*_*out*_(*B*). The dashed curves are the fits to *F*(θ) = *F*(0)/cosθ.

The Dirac fermion behavior probed in our experiments for BaMnSb_2_ is in good agreement with the prediction by first principle calculations that BaMnSb_2_ is a Dirac material at ambient pressure^38^. Next, we will make more detailed comparisons between the predicted electronic band structure and our experimental observations. First, the Dirac bands are predicted to be generated by the Sb square net plane; thus the Fermi surface formed by Dirac bands is expected to be highly 2D, which is supported by our observations. As shown in Fig. 4a,d, systematic evolutions of SdH oscillation patterns for *ρ*_*in*_ and *ρ*_*out*_ are clearly observed as the magnetic field is rotated from the out-of-plane to the in-plane direction (see the insets to Fig. 4b,e for the experiment set-up). The oscillation frequency *F*(θ) extracted from the FFT for *ρ*_*in*_ measurements can be fitted to *F*(θ) = *F*(θ = 0°)/cosθ (Fig. 4c), suggesting that the Fermi surface responsible for SdH oscillations in BaMnSb_2_ is indeed 2D. However, in *ρ*_*out*_(*B,θ*) measurements, we observed two frequency branches. As shown in Fig. 4f, the higher frequency branch also follows *F*(θ) = *F*(0°)/cosθ, while the lower frequency branch shows a weak angular dependence, suggesting the sample used for *ρ*_*in*_ measurements has slightly different morphology in its Fermi surface from the sample used for *ρ*_*out*_ measurements, which presumably originates from slightly different non-stoichiometric compositions. The 2D Fermi surface also explains the aforementioned large electronic anisotropy manifested in the large *ρ*_*out*_/*ρ*_*in*_ ratio (~670). Second, the first principle calculations also predicted that the linear Dirac bands crossing occurs near the middle of ΓM, with the crossing point (*i.e.* the Dirac node) being right above the Fermi level, which implies the Fermi pocket hosting Dirac Fermions should be a hole pocket and small. In addition to the hole pocket enclosing the Dirac nodes, small electron pockets with quasi-linear band dispersion are also predicted to exist at X and Y points. The quantum transport properties of Dirac fermion revealed in our experiments provide strong support to these predictions. As shown in Fig. 3d, our measured Hall resistivity exhibits linear field dependence with positive slopes at all temperatures as well as remarkable SdH oscillations below 50 K. These features prove that holes are dominant carriers and responsible for the SdH oscillations. From the SdH oscillation frequency of 22T of *ρ*_*in*_, the extremal cross-section area of the Fermi surface is estimated to be ~0.2 nm^−2^, about 0.1% of the total area of the first Brillouin zone, indicating an extremely small Fermi surface, the smallest as compared with AMnBi_2_ and SrMnSb_2_ (see Table 2). Third, the SOC-induced gap at the Dirac node in BaMnSb_2_ was predicted to be half of that in SrMnBi_2_ due to the weaker SOC of the Sb square net as mentioned above, which should result in lighter Dirac Fermions in BaMnSb_2_. Our observations of small cyclotron Frequency and effective mass of the Dirac fermions in BaMnSb_2_ are in line with these predicted results. Furthermore, the Dirac cone in BaMnSb_2_ was predicted to be anisotropic^38^, similar to that of SrMnBi_2_ and CaMnBi_2_^23^,^27^,^28^. ARPES experiments are called in to verify it.

The signatures of Dirac fermions in BaMnSb_2_ imply that materials including 2D Sb square net planes can harbor Dirac electrons. We note many such candidate materials indeed exist*, e.g*. ReAgSb_2_ (Re = rare earth), for which small mass quasi-particles have been found^48^,^49^,^50^. Recent studies on LaAgSb_2_ have shown its small mass quasi-particles indeed originate from the Dirac-cone like band structure formed by Sb 5 P_x,y_ orbitals^51^. Band structure calculations^52^ predicted that the Dirac cone in LaAgSb_2_ can host nearly massless Dirac fermions with m^*^ ~0.06*m*_0_ and has a very small Fermi surface with a quantum oscillation frequency of ~20T. However, these predictions were not seen in experiments^48^,^52^; the smallest m* measured in experiments is 0.16*m*_0_ and the least quantum oscillation frequency is 72T^48^. Surprisingly, the Dirac electron behavior observed in BaMnSb_2_ is very close to that predicted for LaAgSb_2_. Note that the electronic structure of BaMnSb_2_ is much simpler than that of ReAgSb_2_. BaMnSb_2_ exhibits only a single frequency quantum oscillations (~22T) and its transport properties can almost be described by a single-band model, whereas LaAgSb_2_ possesses a much complicated band structure, showing four frequencies in quantum oscillations^48^,^49^.

Given that BaMnSb_2_ exhibits a G-type AFM order with possible FM components due to Ba and Mn non-stoichiometry as discussed above, a natural question is whether its FM component can be tuned by changing Ba and Mn non-stoichiometry and coupled to quantum transport properties. In our previous studies on Sr_1-y_Mn_1-z_Sb_2_, we have shown that its saturated FM moment *M*_s_ can be tuned from 0.6μ_B_/Mn to 0.005μ_B_/Mn by changing *y* and *z*^43^. The samples with heavier Sr deficiencies have larger *M*_s_ than the samples with heavier Mn deficiencies. The *M*_s_ (~0.04 μ_B_/Mn at 7T, see Fig. 1c) probed in BaMnSb_2_ seems comparable to that of type B samples of our previously reported Sr_1-y_Mn_1-z_Sb_2_ where *M*_s_ ~0.04–0.06 μ_B_/Mn. Although we have examined many samples, all measured samples show comparable *M*_s_. Therefore, it is difficult to find samples with a wide range of *M*_s_, which would allow us to examine the coupling between Dirac electron behavior and ferromagnetism as we did for Sr_1-y_Mn_1-z_Sb_2_^43^.

## Conclusion

In summary, we have demonstrated that in BaMnSb_2_ the Sb square net layers with coincident stacking of Ba atoms can host nearly massless Dirac fermions due to the weaker SOC effect of Sb, in contrast with massive Dirac fermions hosted by the Bi square net planes in AMnBi_2_. Compared with AMnBi_2_, BaMnSb_2_ displays much more significant 2D-like electronic band structure, with the out-of-plane transport showing non-coherent conduction below 120 K and the *ρ*_*out*_/*ρ*_*in*_ ratio reaching ~670. Its quantum transport properties can be almost described by a single band model, consistent with its simple electronic band structure predicted by first principle calculations. In addition, BaMnSb_2_ also exhibits a G-type AFM order below 283 K and the Ba and Mn non-stoichiometries might cause a weak FM component. These findings establish BaMnSb_2_ as a promising platform for seeking novel exotic properties of massless Dirac fermions in low dimensions.

## Methods

### Single crystal growth and characterization

Single crystals of BaMnSb_2_ were synthesized using self-flux method with a stoichiometric ratio of Ba pieces, Mn and Sb powder. The starting materials were mixed in a small crucible, sealed into a quartz tube under Argon atmosphere and heated up to 1050 °C in one day. The temperature was maintained at 1050 °C for two days. After that, it was first cooled down to 1000 °C at a fast rate, 50 °C/h, and then followed by a slowly cooling down to 450 °C at a rate 3 °C/h. Subsequently the furnace was turned off for fast cooling. Plate-like crystals with lateral dimensions of several millimeters (see the inset to Fig. 1b) can easily be obtained from the final product. The compositions of the crystals were measured using an energy dispersive X-ray spectrometer (EDS). The measured composition can be expressed as Ba_1-y_Mn_1-z_Sb_2_, with *y* < 0.05 and 0.05 < *z* < 0.12. The structure of the single crystals was characterized by an X-ray diffractometer.

### Magnetization and magnetotransport measurements

The magnetization data were taken by a 7T SQUID magnetometer (Quantum Design). The magnetotransport properties were measured using standard four and five- probe method for longitudinal and Hall resistivity, respectively, in a Physics Property Measurement System (PPMS, Quantum Design), and the 31T resistive magnet at National High Magnetic Field Laboratory (NHMFL) in Tallahassee.

### Neutron Scattering

Single-crystal neutron diffraction was performed at the HB-3A Four-circle Diffractometer equipped with a 2D detector at the High Flux Isotope Reactor(HFIR) at ORNL. Neutron wavelength of 1.546 Å was used from a bent perfect Si-220 monochromator^53^. The Rietveld refinement was performed using FullProf^54^.

## Additional Information

**How to cite this article**: Liu, J. *et al.* Nearly massless Dirac fermions hosted by Sb square net in BaMnSb_2_. *Sci. Rep.* **6**, 30525; doi: 10.1038/srep30525 (2016).

## Figures and Tables

**Table 1:** Lattice parameters and atomic positions of BaMnSb_2_ determined from neutron diffraction experiments. The refinement was based on 164 reflections. The R-factor is 0.098 and the *x* ^2^ is 0.33.

T = 4 K				
Space group: *I4/mmm, a* = *b* = 4.4567(54) Å, *c* = 23.85(25) Å Magnetic moment, 3.950(85) μ_*B*_/Mn				
	x	y	z	Site Multiplicity
Ba	0	0	0.11325(55)	4
Mn	0	0.5	0.25	4
Sb-1	0	0.5	0	4
Sb-2	0	0	0.31814(56)	4

**Table 2:** Comparison of the parameters extracted from the quantum oscillations among (Ca/Sr/Ba)MnBi_2_ and (Sr/Ba)MnSb_2_, including the oscillation frequency *F*, reduced effective mass m*/m\_*0*_, extremal cross-section area of Fermi surface *A*_*F*_ (=2π^2^F/Φ_0_), quantum relaxation time τ_*q*_, quantum mobility μ_*q*_ (=eτ_q_/m*), and Berry phase.

	*F* (T)	m*/m_*0*_	*A*_*F*_ (nm^−2^)	*τ*_*q*_ (s)	*μ*_*q*_ (cm^2^V^−1^s^−1^)	Berry’s phase	reference
CaMnBi_2_	101, 181	0.35	0.96, 1.73	—	—	0.9π	25, 26
SrMnBi_2_	152	0.29	1.45	3.5 × 10^−14^	250	1.2π	23
BaMnBi_2_	33, 83	0.105	—	—	—	0.4–0.6π	32
SrMnSb_2_	67	0.14	0.64	4.2 × 10^−14^	570	1.02π	43
BaMnSb_2_	22–35	0.052–0.058	0.21–0.34	3.8 × 10^−14^	1280	1.06π	This work

## References

[b1] Wang Z. *et al.* Dirac semimetal and topological phase transitions in A_3_Bi (A=Na, K, Rb). Phys. Rev. B 85, 195320 (2012).

[b2] Wang Z., Weng H., Wu Q., Dai X. & Fang Z. Three-dimensional Dirac semimetal and quantum transport in Cd_3_As_2_. Phys. Rev. B 88, 125427 (2013).

[b3] Liu Z. K. *et al.* A stable three-dimensional topological Dirac semimetal Cd_3_As_2_. Nat. Mater. 13, 677–681 (2014). 24859642 10.1038/nmat3990

[b4] Liu Z. K. *et al.* Discovery of a Three-Dimensional Topological Dirac Semimetal, Na_3_Bi. Science 343, 864–867 (2014). 24436183 10.1126/science.1245085

[b5] Neupane M. *et al.* Observation of a three-dimensional topological Dirac semimetal phase in high-mobility Cd_3_As_2_. Nat. Commun. 5, 4786 (2014). 24807399 10.1038/ncomms4786

[b6] Borisenko S. *et al.* Experimental realization of a three-dimensional dirac semimetal. Phys. Rev. Lett. 113, 027603 (2014). 25062235 10.1103/PhysRevLett.113.027603

[b7] Weng H., Fang C., Fang Z., Bernevig B. A. & Dai X. Weyl Semimetal Phase in Noncentrosymmetric Transition-Metal Monophosphides. Phys. Rev. X 5, 011029 (2015).

[b8] Huang S.-M. *et al.* A Weyl Fermion semimetal with surface Fermi arcs in the transition metal monopnictide TaAs class. Nat. Commun. 6, 7373 (2015). 26067579 10.1038/ncomms8373 PMC4490374

[b9] Xu S. *et al.* Discovery of a Weyl fermion semimetal and topological Fermi arcs. Science 349, 613 (2015). 26184916 10.1126/science.aaa9297

[b10] Lv B. Q. *et al.* Experimental Discovery of Weyl Semimetal TaAs. Phys. Rev. X 5, 031013 (2015).

[b11] Lv B. Q. *et al.* Observation of Weyl nodes in TaAs. Nat. Phys. 11, 3426 (2015).

[b12] Yang L. X. *et al.* Weyl semimetal phase in the non-centrosymmetric compound TaAs. Nat. Phys. 11, 728–732 (2015).

[b13] Xu S. *et al.* Discovery of a sWeyl fermion state with Fermi arcs in niobium arsenide. Nat. Phys. 11, 748 (2015).

[b14] Xu N. *et al.* Observation of Weyl nodes and Fermi arcs in TaP. Nat. Commun. 7, 11006 (2016). 26983910 10.1038/ncomms11006 PMC4800437

[b15] Borisenko S. *et al.* Time-reversal symmetry breaking Weyl state in YbMnBi_2_. *arXiv:1507.04847v2* (2015). 10.1038/s41467-019-11393-5 PMC6668437 31366883

[b16] Xu Q. *et al.* Two-dimensional oxide topological insulator with iron-pnictide superconductor LiFeAs structure. Phys. Rev. B 92, 205310 (2015).

[b17] Schoop L. M. *et al.* Dirac Cone Protected by Non-Symmorphic Symmetry and 3D Dirac Line Node in ZrSiS. Nat. Commun. 7, 11696, doi: 10.1038/ncomms11696 (2016). 27241624 PMC4895020

[b18] Neupane M. *et al.* Observation of Topological Nodal Fermion Semimetal Phase in ZrSiS. Phys. Rev. B 93, 201104(R) (2016).

[b19] Wu Y. *et al.* Dirac Node Arcs in PtSn_4_. Nat Phys, doi: 10.1038/nphys3712 (2016).

[b20] Bian G. *et al.* Topological nodal-line fermions in spin-orbit metal PbTaSe_2_. Nat. Commun. 7, 10556 (2016). 26829889 10.1038/ncomms10556 PMC4740879

[b21] Hu J. *et al.* Evidence of Topological Nodal-Line Fermions in ZrSiSe and ZrSiTe. Phys. Rev. Lett. 117, 016602 (2016). 27419579 10.1103/PhysRevLett.117.016602

[b22] Liang T. *et al.* Ultrahigh mobility and giant magnetoresistance in Cd_3_As_2_: protection from backscattering in a Dirac semimetal. Nat. Mater. 14, 280–284 (2015). 25419815 10.1038/nmat4143

[b23] Park J. *et al.* Anisotropic dirac fermions in a Bi square net of SrMnBi_2_. Phys. Rev. Lett. 107, 126402 (2011). 22026779 10.1103/PhysRevLett.107.126402

[b24] Wang K., Graf D., Lei H., Tozer S. W. & Petrovic C. Quantum transport of two-dimensional Dirac fermions in SrMnBi_2_. Phys. Rev. B 84, 220401(R) (2011).

[b25] He J. B., Wang D. M. & Chen G. F. Giant magnetoresistance in layered manganese pnictide CaMnBi_2_. Appl. Phys. Lett. 100, 112405 (2012).

[b26] Wang K., Graf D. & Petrovic C. Two-dimensional Dirac fermions and quantum magnetoresistance in CaMnBi_2_. Phys. Rev. B 85, 041101(R) (2012).

[b27] Lee G., Farhan M. A., Kim J. S. & Shim J. H. Anisotropic Dirac electronic structures of AMnBi_2_ (A=Sr, Ca). Phys. Rev. B 87, 245104 (2013).

[b28] Feng Y. *et al.* Strong anisotropy of Dirac cones in SrMnBi2 and CaMnBi_2_ revealed by angle-resolved photoemission spectroscopy. Sci. Rep. 4, 5385 (2014). 24947490 10.1038/srep05385 PMC4064355

[b29] May A. F., Mcguire M. A. & Sales B. C. Effect of Eu magnetism on the electronic properties of the candidate Dirac material EuMnBi_2_. Phys. Rev. B 90, 075109 (2014).

[b30] Jo Y. J. *et al.* Valley-Polarized Interlayer Conduction of Anisotropic Dirac Fermions in SrMnBi_2_. Phys. Rev. Lett. 113, 156602 (2014). 25375728 10.1103/PhysRevLett.113.156602

[b31] Masuda H. *et al.* Quantum Hall effect in a bulk antiferromagnet EuMnBi_2_ with magnetically confined two-dimensional Dirac fermions. Sci. Adv. 2(1), e1501117 (2016). 27152326 10.1126/sciadv.1501117 PMC4846431

[b32] Li L. *et al.* Electron-hole asymmetry, Dirac fermions, and quantum magnetoresistance in BaMnBi_2_. Phys. Rev. B 93, 115141 (2016).

[b33] Wang Y.-Y., Yu Q.-H. & Xia T.-L. Large linear magnetoresistance in a new Dirac material BaMnBi_2_. *arXiv:1603.09117* (2016).

[b34] Cordier G. & Schafer H. Preparation and crystal structure of BaMnSb_2_, SrMnBi_2_ and BaMnBi_2_. Z. Naturforsch. 32b, 383–386 (1977).

[b35] Brechtel E., Cordier G. & Schäfer H. Zur Darstellung und Struktur von CaMnBi_2_. Z. Naturforsch. 35b, 1–3 (1980).

[b36] Guo Y. F. *et al.* Coupling of magnetic order to planar Bi electrons in the anisotropic Dirac metals AMnBi_2_ (A=Sr, Ca). Phys. Rev. B 90, 075120 (2014).

[b37] Wang A. *et al.* Two-dimensional Dirac fermions in YbMnBi_2_ antiferromagnet. *arXiv:1604.01009* (2016).

[b38] Farhan M. A., Lee G. & Shim J. H. AEMnSb_2_ (AE=Sr, Ba): a new class of Dirac materials. J. Phys. Condens. Matter 26, 042201 (2014). 24389589 10.1088/0953-8984/26/4/042201

[b39] He L. P. *et al.* Quantum Transport Evidence for the Three-Dimensional Dirac Semimetal. Phys. Rev. Lett. 113, 246402 (2014). 25541783 10.1103/PhysRevLett.113.246402

[b40] Cao J. *et al.* Landau level splitting in Cd_3_As_2_ under high magnetic fields. Nat. Commun. 6, 7779 (2015). 26165390 10.1038/ncomms8779 PMC4510959

[b41] Narayanan A. *et al.* Linear Magnetoresistance Caused by Mobility Fluctuations in n -Doped Cd_3_As_2_. Phys. Rev. Lett. 114, 117201 (2015). 25839304 10.1103/PhysRevLett.114.117201

[b42] Xiang Z. J. *et al.* Angular-Dependent Phase Factor of Shubnikov-de Haas Oscillations in the Dirac Semimetal Cd_3_As_2_. Phys. Rev. Lett. 115, 226401 (2015). 26650311 10.1103/PhysRevLett.115.226401

[b43] Liu J. Y. *et al.* Discovery of a magnetic topological semimetal Sr_1-y_Mn_1-z_Sb_2_ (y, z < 0.10). arXiv:1507.07978v2 (2015). 10.1038/nmat4953 28740190

[b44] Lifshits E. M. & Kosevich A. M. Theory of the Shubnikov—de Haas effect. J. Phys. Chem. Solids 4, 1–10 (1958).

[b45] Mikitik G. P. & Sharlai Y. V. Manifestation of Berry’s Phase in Metal Physics. Phys. Rev. Lett. 82, 2147–2150 (1999).

[b46] Taskin A. A. & Ando Y. Berry phase of nonideal Dirac fermions in topological insulators. Phys. Rev. B 84, 035301 (2011).

[b47] Xiong J. *et al.* High-field Shubnikov-de Haas oscillations in the topological insulator Bi_2_Te_2_Se. Phys. Rev. B 86, 045314 (2012).

[b48] Myers K. *et al.* de Haas–van Alphen and Shubnikov–de Haas oscillations in RAgSb_2_ (R=Y, La-Nd, Sm). Phys. Rev. B 60, 13371 (1999).

[b49] Bud’ko S. L. *et al.* Thermal expansion and magnetostriction of pure and doped RAgSb_2_ (R=Y, Sm, La) single crystals. J. Phys. Condens. Matter 20, 115210 (2008). 21694227 10.1088/0953-8984/20/11/115210

[b50] Arakane T. *et al.* Electronic structure of LaAgSb_2_ and CeAgSb_2_ studied by high-resolution angle-resolved photoemission spectroscopy. J. Magn. Magn. Mater. 310, 396–398 (2007).

[b51] Shi X. *et al.* Observation of Dirac-like band dispersion in LaAgSb_2_. Phys. Rev. B 93, 081105 (2016).

[b52] Wang K. & Petrovic C. Multiband effects and possible Dirac states in LaAgSb_2_. Phys. Rev. B 86, 155213 (2012).

[b53] Chakoumakos B. C. *et al.* Four-circle single-crystal neutron diffractometer at the High Flux Isotope Reactor. Journal of Applied Crystallography, 44, 655–658 (2011).

[b54] Rodríguez-Carvajal Juan Recent advances in magnetic structure determination by neutron powder diffraction. Physica B 192, 55–69 (1993).

